# Open Access for the Annals of Noninvasive Electrocardiology

**DOI:** 10.1111/anec.12737

**Published:** 2020-01-09

**Authors:** Wojciech Zareba

**Affiliations:** ^1^ University of Rochester Medical Center Rochester NY USA

In year 2020, the Annals enters 25th year of publishing scientific manuscripts: original papers, review articles, and case reports focused on noninvasive electrocardiology. During recent years, we have witnessed transformation of interests toward digital computerized assessment of ECG recordings and increasing use of innovative technologies monitoring ECG signal using different devices such as watches, patches, and other monitoring systems. Our journal became digital few years ago, and now in 2020, we enter the new era of Open Access journal in the spirit of easy access and widespread dissemination of scientific content of the journal. Starting from 2020, all papers published in the Annals will be fully accessible to readers all over the world following current increasing trends of widespread and unrestricted public access to scientific information.

A publication fee, paid for by the authors, is required to facilitate open access publishing, and this approach might limit some submissions. We believe that good quality manuscripts resulting from grants and other funded projects will be submitted and published in the Annals continuing our service to scientific cardiology community.

Advances in electrocardiology requires further attention, and in 2020, we are introducing a new section called New Technologies for which we envision publishing papers on new methods of ECG recording, innovative signal processing, new interpretation, and increasing use of machine learning. We welcome clinical and engineering manuscripts from academia and industry to disseminate further and faster innovations in electrocardiology. In this issue of the journal, we publish two interesting New Technologies papers, one from France on the assessment of signal quality measured with a smart 12‐lead ECG acquisition T‐Shirt (Fouassier, Roy, Blanchard, & Hulot, 2019) and another from The Netherlands on the diagnostic accuracy of a single‐lead portable ECG device for measuring QTc prolongation (Bekker, Noordergraaf, Teerenstra, Pop, & van, 2019). We are very excited about giving this opportunity to academia and industry researchers to share their ideas and opinions about new technologies.

Other interesting papers include very up‐to‐date review on ECG characteristics in patients with heart failure and normal ejection fraction from UK (Nikolaidou et al., 2019) and comprehensive review paper on heart rate variability during endurance exercise from Germany (Gronwald & Hoos, 2019). Original papers worth attention include study on the association between metabolic syndrome and QRT angle from the United States, (Delhey, Jin, Thapa, & Delongchamp, 2019) paper on the association between morphology of left descendent coronary artery and ECG in ST‐elevation myocardial infarction from Japan (Fujii, Hasegawa, Nakamura, & Ikari, 2019), study on temporal changes of noninvasive electrocardiographic risk factors for sudden cardiac death (Xenogiannis et al. 2019), and a paper on compound and heterozygous KCNQ1 mutations in LQTS from China ( Lin et al. 2019).

At the beginning of the year, we would like to thank all authors for submitting manuscripts and for citing papers published in the Annals. Very special thanks go to numerous reviewers of paper submitted to the journal. List of reviewers is shown below. We are looking forward to advancing the field of electrocardiology, clinical cardiology, and new technologies with your help.

Wishing you Successful New Year with the Annals at your side!
